# Supplementary material for: Arbuscular Mycorrhizae Alter Photosynthetic Responses to Drought in Seedlings of Artemisia tridentata
Source: Plants (Basel). 2023 Aug 19;12(16):2990. doi: 10.3390/plants12162990 (PMC10458374; doi:10.3390/plants12162990)
Supplement: Supplementary file 1 [file plants-12-02990-s001.zip › Supplementary Tables&Figures.pdf]

Table S1. Percent colonization of *Artemisia tridentata* roots by septate fungi before and after the imposition of drought. Inoculated plants were grown in a potting mix supplemented with arbuscular mycorrhizal spores. Medians (and 95% confidence limits) of five plants. Plants sampled before the drought were different from those after the drought. The *p*-values are based on unpaired Wilcoxon tests.

Colonization	Non-inoculated	inoculated	*p*-value
Total before	6.8 (4.2, 9.3)	5.0 (1.4, 8.7)	0.262
Microsclerotia before	2.5 (0.6, 5.0)	0 (0, 2.5)	0.065
Total after	2.2 (0.8, 6.4)	0.8 (0.5, 4.0)	0.690
Microsclerotia after	2.2 (0.8, 3.7)	0 (0, 2.7)	0.095

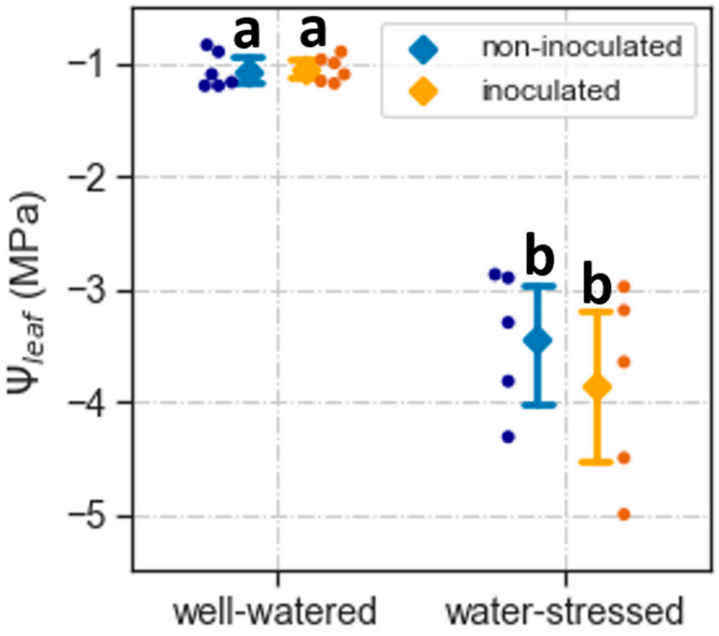

Figure S1. Water potential of well-watered and water-stressed *Artemisia tridentata* seedlings. Circles show the water potential of individual seedlings, while diamonds and error bars represent means and 95% confidence limits. Based on Welch's ANOVA test, different letters indicate

statistical differences ($p < 0.05$). In water-stressed seedlings, plant water potential was determined after stomatal closure ($g_s < 0.03$ mol m^{-2} s^{-1}).

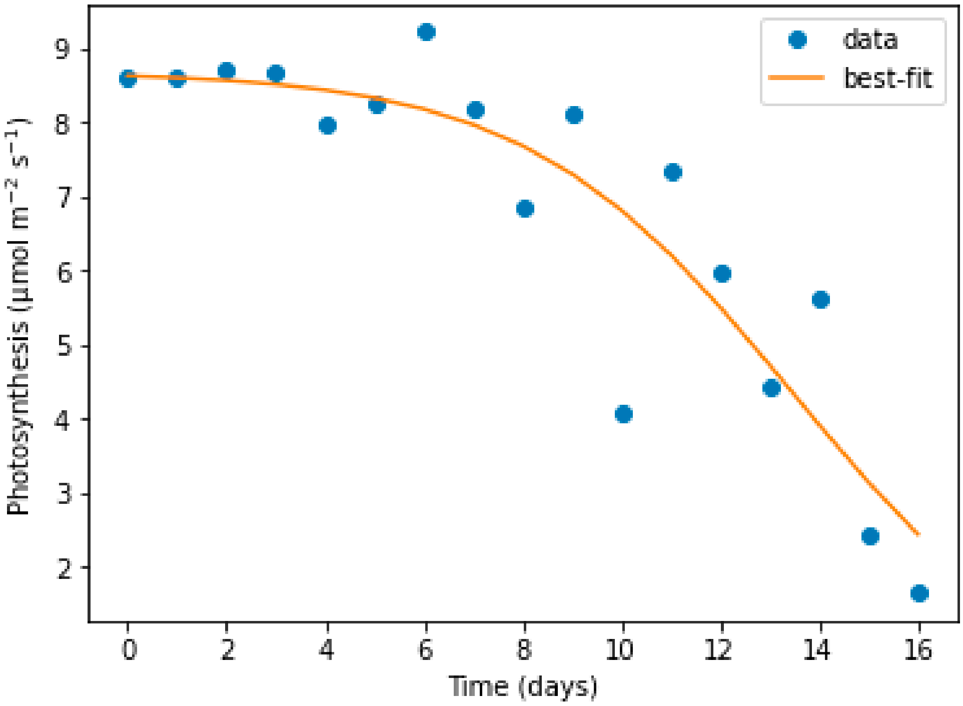

Figure S2. Example of the time course of changes in CO_2 assimilation during the progression of drought. For this plant, the value of *I*, *S*, and t1/2 was 8.69 μmol CO_2 m^{-2} s^{-1}, 0.37, and 13.4 days, respectively.

Table S2. Percent colonization of *Artemisia tridentata* roots by septate fungi before and after the imposition of drought, and in plants always kept well-watered. Inoculated plants were grown with soil and roots from trap cultures. Medians (and 95% confidence limits) of 4 to 6 plants. Plants sampled before the drought were different from those after the drought. *P*-values from unpaired Wilcoxon tests.

Colonization	Non-inoculated	inoculated	*p*-value
Total before	0.8 (0, 8.6)	8.3 (4.5, 11.4)	0.060
Microsclerotia before	0 (0, 1.8)	1.8 (0, 6.4)	0.310
Total after	19.4 (0.8, 59.0)	21.0 (15.6, 72.8)	0.329
Microsclerotia after	0.6 (0, 6.2)	0 (0, 6.1)	0.662
Total well-watered	34.2 (22.3, 47.9)	19.3 (5.7, 73.8)	0.686
Microsclerotia well-watered	4.3 (0, 18.8)	1.6 (0, 10.8)	0.486

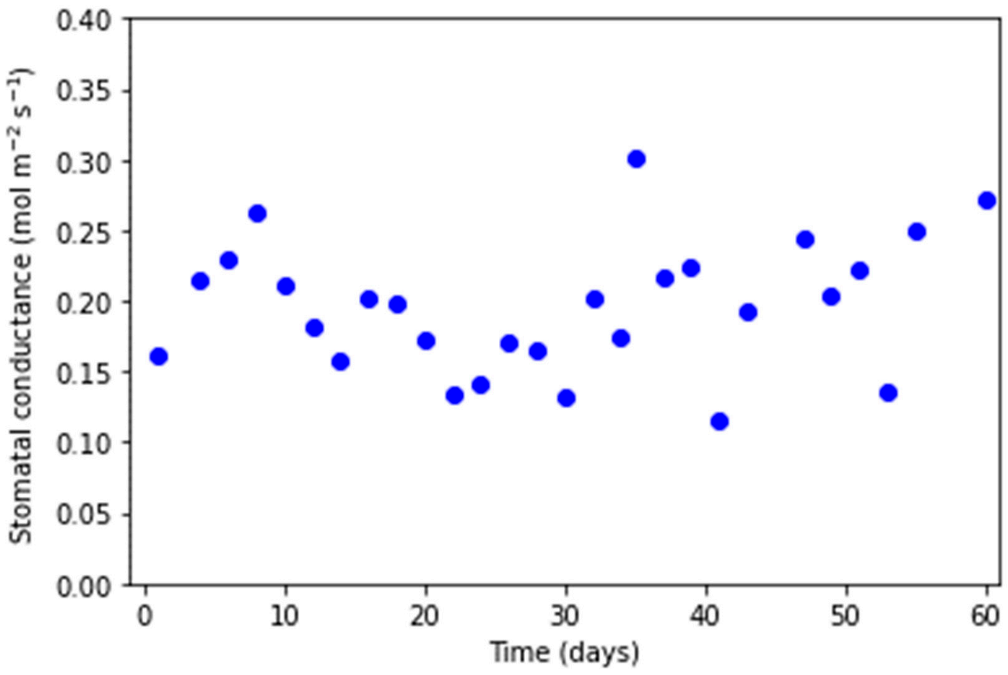

Figure S3. Example of daily variations in stomatal conductance observed in well-watered plants. Similar patterns were observed for CO_2 assimilation, leaf transpiration, and the operating efficiency of photosystem II.

Table S3. Comparison of stomatal conductance (g_s), CO_2 assimilation (A), leaf transpiration (tr_{leaf}), and operating efficiency of photosystem II ($\Phi PSII$) in non-inoculated and inoculated *Artemisia tridentanta* plants growing under well-watered conditions. Mean (±SE) of 4 plants; each plant was measured 30 times at 2d intervals over 60 days. *P*-values based on t-tests

Parameter	Non-inoculated	inoculated	*p*-value
A (µmol CO_2 m^{-2} s^{-1})	11.4 (±0.6)	10.6 (±0.7)	0.38
tr_{leaf} (mol H_2O m^{-2} s^{-1})	4.8 (±0.4)	5.1 (±0.1)	0.48
g_s (mol H_2O m^{-2} s^{-1})	0.24 (±0.03)	0.25 (±0.01)	0.75
$\Phi PSII$	0.18 (±0.01)	0.17 (±0.01)	0.33

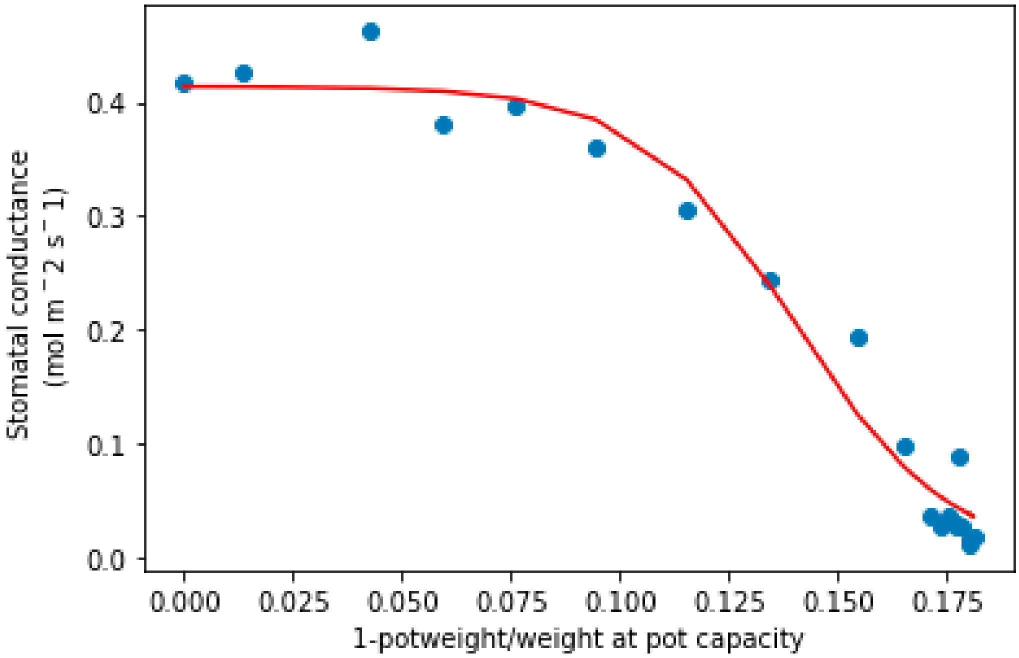

Figure S4. Example of changes in stomatal conductance as the soil dries out. For this plant, the value of *I*, *S*, and $rPC_{1/2}$ was 0.41 mol H_2O m^{-2} s^{-1}, 56.9, and 0.86, respectively.

Table S4. *P*-values from the two-way ANOVA of the colonization results from the field experiment

	Inoculation	Sampling time	Inoculation x sampling time
Total AMF colonization	0.0004	0.020	0.759
Arbuscules	0.0188	0.106	0.615
Vesicles	0.316	0.602	0.191
Total septate fungi colonization	0.103	0.501	0.679
Microsclerotia	0.168	0.222	0.999

Table S5. Percent colonization of field-grown *Artemisia tridentata* plants by septate fungi. Inoculated plants were supplemented with soil and roots from trap cultures at transplanting. Plants were harvested eight (spring) or twelve (fall) months after outplanting- Average and standard errors of eight (spring) or four (fall) plants.

Colonization	Non-inoculated	inoculated	*p*-value
Total spring	32.5 (±4.9)	43.9 (±4.9)	0.38
Microsclerotia spring	8.9 (±3.3)	14.3 (±3.3)	0.65
Total fall	30.9 (±7.0)	37.2 (±7.0)	0.91
Microsclerotia fall	3.8 (±4.7)	9.3 (±4.7)	0.84

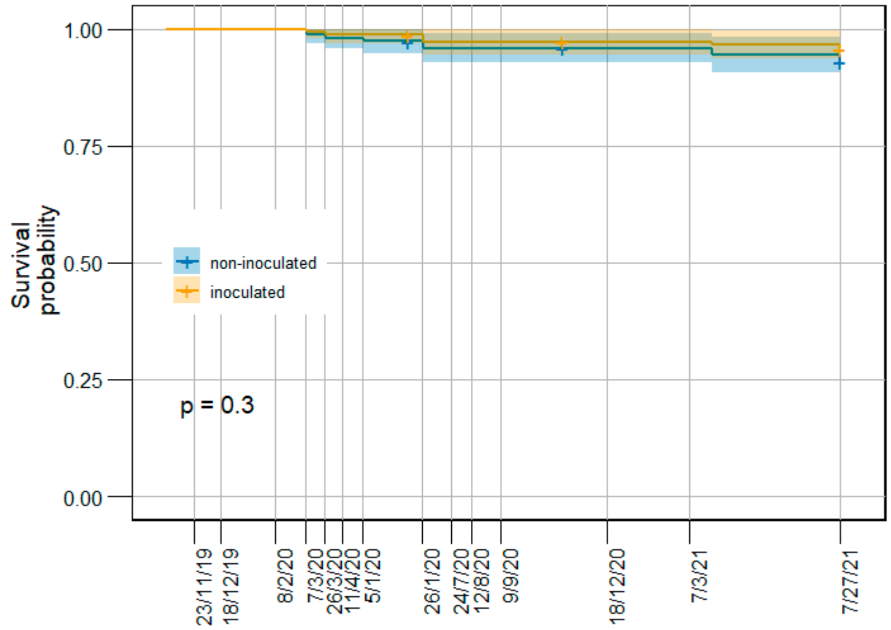

Figure S5. Survival of non-inoculated and inoculated *Artemisia tridentata* seedlings during the first two years after outplanting. Survival curves indicate median survival and 95% confidence intervals; *p*-value based on a log-rank test. The dates indicate when survival was measured.

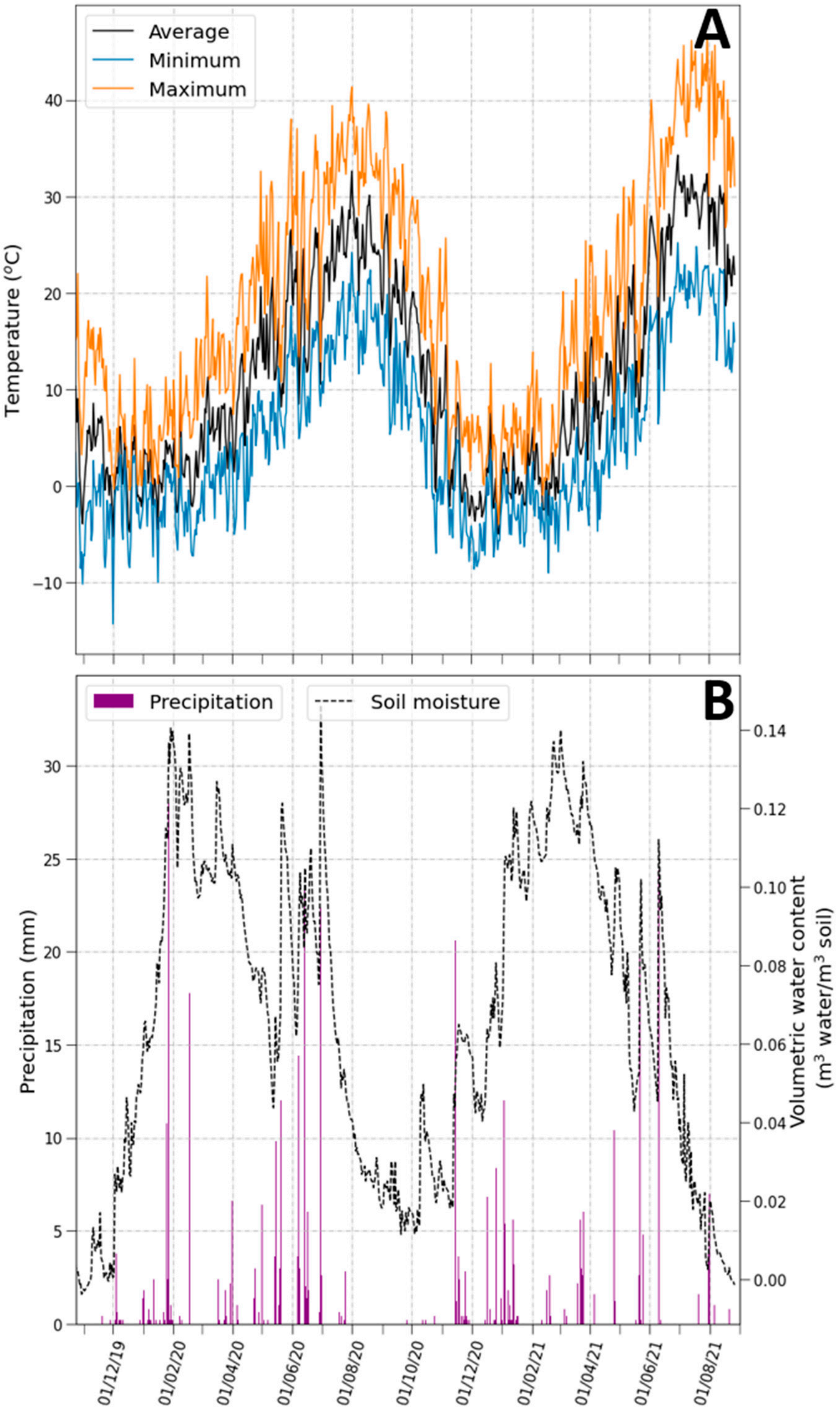

Figure S6. Weather conditions during the experimental period. **A**, Temperature, **B**, Soil moisture in the upper 20 cm of the soil and precipitation.
